# Progress of the Medical Sciences

**Published:** 1919-12

**Authors:** 


### Progress of the Medical Sciences.

### SOME ASPECTS OF MODERN CHEMO-THERAPEUTICS.

### A SUMMARY OF LECTURES BY DR. M. NIERENSTEIN.

UNDER this title Dr. M. Nierenstein, Lecturer in Bio-Chemistry to the University of Bristol, has given a course of lectures, summarised here by his kind permission. For some years he has been working (under the direction of Sir Ronald Ross) at the chemistry of the action of drugs on parasites, and this course of lectures gives a brief account of some of his work.

After a remark on the fact that parasiticidal drugs act on the host as well as on the parasite, the first lecture dealt with the action of atoxyl on trypanosomes. If atoxyl is injected into a rat infected with trypanosomiasis the trypanosomes soon show a lessened activity. Seven hours after the injection they are no longer found in the rat's blood. Soon after this the animal loses the symptoms of trypanosomiasis. Yet other rats inoculated with his blood often become infected with trypanosomiasis. After a period which varies with the strain of trypanosome the first rat has a relapse. Suppose this again treated with atoxyl; once more the symptoms disappear, yet the rat's blood is once again found to be infective when inoculated into other rats. As this process is repeated the intervals between the relapses grow shorter, until they practically disappear. The strains secured by inoculation from the original rat into others also become more and more virulent, and at the same time undergo definite modification of structure. Similar structural change can be produced by passing mammalian trypanosomes into birds and then back into mammals. In each case, whether by animal transmission or by habituation to atoxyl, the parasites are made "tougher."

Experiments by Dr. Nierenstein and others suggest that the drug does not act on the parasite direct, but only with the help of the host's serum, this latter playing intermediary between drug and parasite much as a mordant does between dye stuff and fabric.

The atoxyl inoculations have, then, produced a trypanosome which is resistant to or immunised against atoxyl. This immunity is retained by the parasite during transmission through animals of the same species as that in which the immunity is acquired. So long as rat is inoculated from rat the immunity holds; but if the trypanosome is passed from rat to rabbit it loses its immunity to atoxyl. Even transmission to the louse destroys the immunity.

Whatever the explanation of these phenomena, three facts are certain: (1) the parasite has been immunised against atoxyl by repeated exposure to that drug in the presence of blood serum; (2) the immune parasite has an enhanced virulence; (3) immunity is lost by transmission through an intermediate host. If these facts were to prove equally applicable to the action of salvarsan on the spirochæta pallida present-day plans for the stamping out of syphilis would need a good deal of modification. Even the development of quinine-resistant plasmodia in malaria is not quite so serious a matter, because passage through an intermediate host, the mosquito, would serve to rob the plasmodium of its immunity.

In the second lecture the relation between chemical constitution and physiological action was discussed. That such a relation exists is well known, but what are its laws? Observations by Dr. Nierenstein and others on the excretion of quinine confirm the belief that there is a constant relation between the concentration of a drug in the blood and that in the urine. Attempts to override this law and saturate the blood with a drug are apt to injure the kidneys and to fail in their object. Supersaturation of the body with quinine was followed, not only by increase in the amount of quinine in the urine, but also by albuminuria. This observation led to a consideration of the means which the body uses in getting rid of drugs. Substances of the caffein group, for example, are merely worked out of the body by an increase in the outflow of urine, a fact which recalls their derivation from urea. Others, such as quinine, undergo oxidation, partial or complete; while others again are altered by addition of substances more complex than oxygen. Carbolic acid, for instance, combines with sulphuric acid to form an excretion product.

The next point was the well-known alteration of physiological action effected by even small changes in chemical constitution. Various examples were quoted by the lecturer, among them the conversion of carbolic into salicylic acid. In 1912 Dr. O. C. M. Davis, of Bristol, compared the chemical activity of a number of the aniline derivatives with their toxicity, and found a definite parallelism. Substances which show strong affinities in the test-tube show them also in the body.

The lecturer alluded to the highly suggestive relation existing between molecular asymmetry and physiological action, quoting Crum Brown's work, which shows that asymmetric compounds of nitrogen, arsenic, antimony, sulphur and even cobalt are alike in exerting a curare effect after injection into the body.

In his third lecture Dr. Nierenstein traced the evolution of the modern organic compounds of arsenic, from cacodyl to salvarsan. This evolution, which aimed at increasing the physiological and therefore the therapeutic activity of the arsenical combination, included two decisive steps. The first was that in which it was determined to combine arsenic with aniline rather than with one of the fatty acid series, presumably because greater physiological activity was expected of the aniline-arsenic compound. In the second, Ehrlich conceived the idea of using chemically unsaturated arsenic compounds, the underlying idea being a comparison between the physiological activity of the unsaturated carbon monoxide and the saturated carbon dioxide. It was thus that he arrived at salvarsan and its more soluble derivative neo-salvarsan. Finally, others have combined copper, silver and other antiseptics with salvarsan, a latter-day equivalent of the ancient " buck-shot " prescription. Indeed, one cannot but be struck by the thought that throughout this development of a complex arsenical molecule the same spirit of empiricism that prompted the older physicians to try a drug and see what happened has been at work, albeit under the guise of scientific infallibility.

In the fourth lecture certain elaborations of the idea of synthesising compounds for therapeutic purposes were examined. First, the steps adopted for using antimony in organic compounds, instead of or in addition to arsenic, were briefly described. The rationale of this proceeding is, of course, to be found in the atomic similarities between arsenic and antimony. After showing that the use of antimony had on the whole been disappointing, the lecturer asked why more attention had not been concentrated on phosphorus, an element lying midway between nitrogen and arsenic. He pointed to the fact that phosphorus is normally present in protoplasm, which suggests that its compounds might be more easily assimilated than those of arsenic. He thought that research in this direction, however laborious, could not properly be neglected.

Second, experiments with dyes were summarised. These chiefly concerned two groups of substances, the diazo compounds and the triphenyl-methane series. The attempt to apply these substances to the killing of parasites was prompted by their known and visible union with the protoplasm of the parasites. Briefly, the results in this direction are disappointing.

The last lecture was in many respects the most interesting of all ; partly because it was based almost entirely on Dr. Nierenstein's recent work, partly because its subject, the parasiticidal action of quinine, is one which must always have a deep interest for British medicine. The first point made was the chemical relation between the three principal alkaloids of cinchona. Cinchonine is the simplest and least parasiticidal. A small addition to its molecule converts it into cuprein, which is rather more powerful in its action on plasmodia. A further small addition brings us to quinine, the most active of all. By a further expansion of the molecule the compound ethyl-hydrocuprein is formed, which has no effect on the malaria parasite, but inhibits the growth of pneumococci.

The lecturer next passed to a consideration of the fate of quinine within the body. Its excretion as such in the urine was first definitely proved about sixty years ago by Dr. Herapath of the Bristol Medical School. The test which he devised is, as Dr. Nierenstein's researches showed, still the most reliable known. By its use traces of 1 in 15,000,000 can be detected. Approximately 50 per cent. of the quinine taken is excreted as such. The method by which it is administered does not alter this, neither does it influence the rate at which the drug is excreted.

As to the fate of the remainder of the quinine taken not much is known. Dr. Nierenstein discovered two disintegration products, quitinine and hæmoquinic acid, in the urine, and he is inclined to think that the action of the drug in malarial plasmodia is exercised by substances similar to these and not by quinine itself. The quinine molecule has no amino group or other physiologically active radicles. Hæmoquinic acid has not yet been tested, but quitinine (which Ramsden of Liverpool has shown to be formed by oxidation of quinine by means of liver emulsion) has no effect on malaria. There are, however, substances which have been synthetically prepared which have an action on malaria. The molecule of quinine itself has not yet been synthesised, but these synthetic products represent a stage in the synthesis of quinine; the molecule of each is a part of the molecule of quinine.

There may well be a future for bio-chemical research into the action of quinine. It might be possible to improve upon the therapeutic effect of quinine, either by the introduction of amino groups into its molecule or by synthesis. If the synthetic compounds referred to above act on the malaria plasmodium, and if quinine itself acts by virtue of metabolising into something not unlike those compounds, it might be possible to hit upon a molecule which had all the anti-malarial properties of quinine. To give such a compound would be more economical than giving quinine, because all of it would act, and none would pass through the body inactive, as appears to be the case with a large portion of the quinine administered.

Such a research would provide a field for that co-operation between clinician and scientist which was advocated in this year's Presidential Address to the Medico-Chirurgical Society. And it would furnish an interesting and highly significant contrast to the search for an arsenical spirillicide already alluded to in these notes. It would take account of the fact that quinine is good for the treatment of malaria, whereas the researches that culminated in salvarsan appear to have ignored in an indefensible degree the proved value of mercury in the treatment of syphilis. Possibly this mistake might have been avoided if there had been a closer collaboration between chemist and clinician.

CAREY COOMBS.

---

### FOURTH INTER-ALLIED SURGICAL CONFERENCE FOR THE STUDY OF WAR WOUNDS.

(*Paris, October*, 1919.)

### CONCLUSIONS ADOPTED ON THE VARIOUS QUESTIONS SUBMITTED FOR DISCUSSION.

**Immunisation.**—Amongst wound infections tetanus and gas gangrene have been effectively dealt with by means of immunisation. The immunisation has been obtained by serum therapy. [1]

**Tetanus.**

I. PREVENTIVE SERUM THERAPY.

A. The experience of the war has cofirmed the preventive value of anti-tetanic serum. The records show that :

B. Tetanus almost completely disappeared after the first months of the war.

C. This diminution was due above everything else to preventive serum therapy.

D. Improvements in surgical treatment played their part in this result.

E. Serum should be injected as early as possible after the wound is inflicted.

(*a*) In the French and Belgian armies after the initial injection a second injection is usually given on the 7th to 10th day after wounding. The routine dose consists of 4,000 antitoxic units [2] contained in 10 c.c. of serum.

(*b*) In the British and Portuguese armies the following procedure is recommended : after the initial injection prophylactic injections are repeated every seven days so long as the wound remains open.

It is unnecessary to go beyond four doses. Each injection consists of 500 antitoxic units (U.S.). [3]

(*c*) In the Italian army every case was given two injections of A.T.S. at twenty-four hours' interval. In certain severe cases a third injection was given about the 7th day.

(*d*) In the American Army (U.S.) a single injection of 1,500 (U.S.) units of A.T.S. was recommended, to be given as early as possible after wounding. The injection was not repeated unless symptoms of tetanus appeared or later operations had to be performed.

G. Any later operation renders a further injection necessary.

E. When, as rarely happened, tetanus developed in spite of prophylactic inoculation, its clinical severity was often very mild.

II. CURATIVE TREATMENT, CURATIVE SERUM THERAPY.

A. Serum therapy is innocuous as a curative measure provided the necessary precautions are taken against anaphylaxis on giving intravenous or intraspinal injections.

B. The question of the efficacy of curative serum therapy requires further study.

**Gas Gangrene.**—The conclusions agreed upon at the last session remain unaltered.

**Late Results in Fractures of the Femur.**—The late results as seen during the war varied according to the period of the war in which the fractures of the femur occurred.

1. At the beginning of the war the results were bad. The lack of good pparatus for immediate immobilisation and transport, the inadequacy of treatment and splinting account for these deplorable results.

2. During a second period, which may be reckoned as beginning towards the end of 1916, the end-results improved by reason of the routine disinfection of wounds, the advances made in surgical technique, in the early splinting, and in splinting during later treatment. The institution of special centres had, from this point of view, the best effect.

3. During active fighting, even in this second period, the results were often less satisfactory than in quiet times. This was due not to defective surgical methods, but to the inrush of wounded, which prevented the application of these methods fully and vigorously.

We have not actually sufficient data to enable us to determine the proportion of late complications following fracture of the femur, that is to say, shortening, deformities, osteomyelitis, interference with the action of the knee-joint, of the hip or foot; but the decrease in the number and in the severity of these complications during the last two years of the war justify the following conclusions :—

Disinfection of the site of fracture by operation or chemical methods, followed by primary or secondary suture, diminished considerably the number of cases of osteomyelitis and its sequels. Reduction of fractures of the shaft by extension is the method of choice, provided that it is always combined with early movement of the joints. Every effort of the surgeon should be directed to averting ankylosis or stiffness of the knee, which appears to be still a too common sequel. For infra-trochanteric fractures extension in abduction gives the best results.

In fractures of the lower third and near the condyles which cannot be reduced by continuous extension, direct traction on the condyles or osteo-synthesis are the methods which appear to lead to the most satisfactory anatomical and functional results.

Faulty unions with angular deformity or rotation which have been treated by osteotomy, either with or without osteo-synthesis have in many cases been improved. In every case coming under observation early movements, active or passive, should be carried out.

**Surgical Treatment of Scars.**—Only scars of the limbs and trunk have been reported upon.

A. Preventive Treatment.—Other things being equal, the great cause of vicious scarring is suppuration of wounds. Preventive treatment of infection in war wounds consists of anatomical repair of the region by primary suture, which at the same time constitutes the preventive treatment of vicious scarring.

B. Curative Treatment.—In closed scars, either contracted or adherent, physiotherapy (*i.e.* treatment by electricity, massage movements, hot baths, ionisation, radium therapy, etc.) patiently persisted in is capable of yielding excellent results. It should be replaced by operative treatment when no further benefit is produced, or when the cicatrix is old.

(*a*) *Operative Treatment.*—In contracting scars (especially those that involve the superficial planes) the whole surface and depth of the scar must be excised, the skin edges must be brought together and sutured without tension. This can be rendered possible by free mobilisation of the edges of the wound obtained according to the nature of the case either by subcutaneous undercutting, by autoplasty, or by progressive distension of the skin following Morestin's method.

Speaking generally, grafts of any sort give results inferior to autoplastic methods.

In adherent deep scars involving muscles, tendons, or bone excision of the scar *en bloc* should be performed, and the various anatomical planes (muscles, aponeurosis, skin) of the region reconstructed by suture of each separately.

Where there has been extensive destruction of tendons different methods may be adopted, particularly the use of hetero-plastic grafts (methods of Nageotte-Sencert). The operation may be long and difficult, and it may be advisable to do it in several stages.

Ulcerated scars which cannot be excised may be dealt with by incisions round the circumference of the scar or by Thiersch grafts.

(*b*) *Post-operative Treatment.*—Physiotherapeutic treatment at a subsequent stage is recommended in most cases.

**Late Results of Joint Wounds.**

1. Punctured bullet wounds of joints which have not been operated on often (the proportion cannot be determined) cause fixation and grating of the joint. This grating is produced by cartilaginous injuries (velvety conditions), without direct connection with the track of the projectile.

2. Lateral arthrotomy, with suture, has given after long interval very good results. Transtendinous arthrotomy has given much less satisfactory results, and is generally followed by limitation of movements. Sometimes after lateral arthrotomy a considerable laxity of the joint occurs, apparently due to prolonged distension by sero-sanguineous effusion.

3. Primary resection performed strictly according to Ollier's sub-capsular periosteal method has given good functional results as regards firmness and mobility. Remarkable bony regeneration generally follows.

4. Resection whilst the pyrexia continues (by Ollier's method) generally gives good functional results.

5. As regards the value of immediate active movements in the treatment of non-infected joint wounds and in purulent arthritis the conclusions adopted at previous conferences should be referred to. The functional results have been maintained over long period, and in certain cases further improvement is seen quite late.

**The late effects of Chest Wounds.**—These must be considered separately from the surgical and medical point of view.

A. SURGICAL.—(*a*) *Small Wounds, Closed Thorax, without Surgical Intervention.*—The degree of recovery of function depends principally upon the resolution of the hæmothorax.

If the effusion is *limited* and aseptic, there may be perfect recovery. Non-infected hæmothorax, which is abundant and persistent, leaves anatomical and functional changes in the shape of pleuro-pulmonary adhesions, which form particularly in the costo-diaphragmatic cul-de-sac.

With a retained missile absolute tolerance may exist; on the other hand, functional troubles which are occasionally serious, or even delayed infection, may supervene.

(*b*) *Wounds requiring Immediate Operation.* Complete immediate thoracotomy usually gives good late results. The more limited the bony injury the better the result. From this point of view large flaps are very inferior to limited rib-resection as a mode of access to the thorax.

(*c*) *Wounds requiring Secondary Operation for Infection.*—Operations for empyema or infected hæmothorax have proved in their anatomical and functional results far less satisfactory than immediate operations. Thoracic function is the more completely restored according as the bony interference for drainage has been the less extensive and the cure most rapid.

Methods of pleural sterilisation (Tuffier-Depage), allowing of secondary suture, have secured functional recovery superior to those which allow of secondary scarring by granulation or extensive costal resection.

In cases of persistent intractable fistula operations for freeing the lung from its fibrous envelope (pleurectomy) have furnished better functional results than extensive thoracoplastics or thoracectomies. The latter should no longer be employed save for exceptional conditions.

(*d*) *Wounds requiring Secondary Operation for any other cause than Infection, especially for Secondary Extraction of Missiles.*—The results of secondary operation for extraction of missiles have as a rule been good.

B. MEDICAL.—1. Late parietal cicatrisation, in proportion to its extent, is a cause of more or less important diminution of functional capacity. Fistulas (bony or pleural) indicate the persistence of an active focus and call for operation.

2. Pleural scars may according to their extent cause varying degrees of interference with respiration. They may give rise to no interference. Encysted collections may form in the course of evolution (remains of hæmatoma or re-infection of an old focus).

3. Pulmonary sequels may also be either definitive or evolutive. Definitive or cicatricial lesions lead to respiratory embarrassment which is often incurable, or to an embarrassment capable of gradual improvement by breathing exercises.

Evolutive lesions occur in the form of local inflammatory foci and encysted effusions, and cystic hæmatomas which may one day suppurate and end by cavity formation.

4. Patients with old chest wounds often complain of pains, dyspnœa and palpitations, sequels which may originate in damage to the mediastinal nerves or the cardiac plexus, even when no missile is retained. One should not hastily suspect malingering or exaggeration even though there is complete absence of radioscopic or stethoscopic signs.

5. The pleuro-pulmonary sequels of chest wounds may often develop into general symptoms (hæmoptysis, dyspnœa, pyrexia, and physical signs) which may be mistaken for tuberculosis. The frequency of post-traumatic tuberculosis has been exaggerated; in reality it is exceptional. A physician's first duty is to avoid mistakes in diagnosis, and therefore never to omit to examine for bacilli. Moreover, in cases of proved tuberculosis it is very difficult to state positively that it was caused by the trauma ; this is a surmise based on the estimate of the time which has elapsed between the trauma and the first appearance of symptoms. Finally, when there has been pre-existing tuberculosis one wants to know whether the trauma has lit up quiescent disease, or aggravated it, or whether the tuberculosis has pursued its normal course.

NOTE.—In the Italian army immediate artificial pneumothorax has been performed to procure hæmostasis and to prevent infection. This method has given satisfactory anatomical and functional results both immediately and subsequently.

**Late Results of Head Wounds.**

I. Cerebral complications following wounds of the skull and brain are not so common as was believed before the war.

II. Secondary abscess of the brain appears to have usually a very definite origin ; it is due in most instances to a projectile or foreign body retained in the brain substance. It became less common directly the treatment was adopted of routine immediate removal of projectiles or foreign bodies, together with thorough cleansing and disinfection of the track followed by primary or secondary suture of the wound.

III. The onset of cerebral abscess is often insidious, it may occur after varying lapse of time, even after a considerable interval.

IV. Late meningo-encephalitis is equally rare ; occasionally it may develop suddenly.

V. Of all complications of head wounds epilepsy seems the commonest and the most grave (10 per cent. of cases).

VI. It is frequently of a generalised character from the outset, more rarely it assumes the Jacksonian type.

VII. It is often difficult to determine the cause of the complications or the site of the lesion which excites them.

The general conclusions, especially those relating to secondary operations, confirm those of the 1917 Conference.

**Late Results of Wounds of the Spinal Cord.**—1. The late results of spinal-cord wounds can only be observed in a limited number of cases because the prognosis of such wounds is very grave in the early stages. The mortality in the first few days or weeks was about 60 per cent. in the ambulances of the armies.

2. Complete division of the cord by bullet or shell fragment is almost without exception fatal within a few days or weeks. In such cases as survive phenomena of automatism can be recognised in the lower segment of the cord.

3. Cases surviving partial division of the cord, myelomalacia, or hæmatomyelia often remain permanent paraplegics. Some become bed-ridden with sloughy sores, œdema, and urinary infections, others retain the power of certain movements and can walk with crutches, while others make even further progress. The prognosis depends also upon the extent of the lesion in length and depth.

4. Lesions involving only half the cord with Brown-Sequard syndrome and transverse posterior hemisections of the cord are less serious in prognosis, and improvement has often been observed.

5. Paraplegic symptoms due to simple compression of the cord by hæmatorachis or sub-arachnoid hæmorrhages round the cord are capable of improvement, even the pain due to irritation of the nerve-roots by bony compression may be cured by removal of the compressing fragments.

6. The prognosis in injuries of the *Cauda equina* is less serious than in those of the cord. Cases that do not die quickly of meningeal complications often improve in the course of weeks or months.

7. Paraplegia due to concussion of the spinal cord, and to hæmatomyelia without lesion of the dura mater has a better prognosis than that due to destructive changes caused by projectiles which have penetrated the dura mater. The same remark applies to hæmatomyelia due to the explosive effects of H.E. without external wounds.

The gravity of the prognosis in spinal-cord wounds depends on meningeal, pulmonary, vesical and renal complications, on bed-sores, on derangements of the sympathetic innervation of the abdominal viscera and endocrine glands, on bulbar anæmia and on organic defects which predispose to terminal infections. Finally, it must be borne in mind that paraplegics who survive often remain from the social point of view confirmed invalids.—Reprinted from *La Presse Medicale*, Nov., 1919.

### REFERENCES.

[1] In the Italian army injection of anti-diphtheritic serum has given most satisfactory results in cases of diphtheritic infection of wounds.

[2] The French antitoxin unit represents the quantity of serum required to counteract 100 fatal doses of toxin. A fatal dose of toxin is calculated as the amount necessary to kill a guinea-pig weighing 350 grammes in 4½ to 5 days after inoculation into the muscles of the thigh.

[3] The United States antitoxin unit according to Rosneau's definition represents ten times the quantity of serum required to counteract 100 minimal fatal doses. A minimal fatal dose is calculated as the smallest quantity which injected subcutaneously will kill a guinea-pig weighing 350 grammes in four days.

